# Determination of selected heavy metals in soil and their impact on *coffee* bean composition, in case of West Guji Zone, Ethiopia

**DOI:** 10.1016/j.toxrep.2026.102323

**Published:** 2026-08-08

**Authors:** Yakob Geda Debeli, Tola Jabessa Masho, Gemechu Duguma Argessa, Mizbawu Kenea Gurmu, Dereje Abdisa Saketa, Kenenisa Abdisa Kuse

**Affiliations:** a Department of Chemistry, College of Natural and Computational Sciences, Bule Hora University, PO Box 144, Bule Hora, Ethiopia; b Department of Industrial Chemistry, College of Natural and Computational Sciences, Bule Hora University, Bule Hora, Ethiopia; c Deparment of Environmental Engineering, College of Engineering, Bule Hora University, Bule Hora, Ethiopia; d Department of Statistics, College of Natural and computational Sciences, Bule Hora University, Bule Hora, Ethiopia

**Keywords:** Heavy metals, soil-plant transfer, Coffee bean, health risk assessment, FAAS, Ethiopia

## Abstract

*Coffee (Coffea arabica **L**.)*is one of the most important agricultural commodities in the world and a major source of income for millions of farmers, with Ethiopia recognized as a center of origin and genetic diversity. Despite the economic importance of specialty coffee produced in the western Guji area, limited information is available on the contamination of coffee-growing soils with heavy metals, the transfer of metals from the soil to the beans and the associated risks to human health. The study measured concentrations of selected heavy metals (Cu, Mn, Zn, Ni, Co, Cr, Cd and Pb) in coffee-growing soils and green coffee beans sampled from the districts of Hambela, Bule Hora and Kercha, and assessed their transport from the soil to the bean. The samples of the soil and coffee beans were digested by an optimized wet acid digestion process and analyzed by Flame Atomic Absorption Spectrometry (FAAS). The analytical method demonstrated excellent linearity (R² = 0.9985–0.9989), good response rates (87–115 mg L⁻¹), and low detection limits (RSD < 5.38 mg). The concentrations of Cu, Mn, Zn, Ni, Co, and Cr in Coffee beans ranged from 20.10 ± 1.30–22.44 ± 1.70, 14.25 ± 1.20–17.14 ± 1.30, 6.74 ± 1.10–8.43 ± 1.10, 1.22 ± 0.20–1.43 ± 0.30, 1.47 ± 0.30–1.49 ± 0.20, and 0.08 ± 0.01–0.92 ± 0.10 mg/kg, respectively, whereas Cd and Pb were below the method detection limits. Soil samples contained comparatively higher metal concentrations, particularly Mn (237.11 ± 3.30–285.44 ± 6.30 mg/kg) and Cu (59.40 ± 2.30–64.40 ± 2.50 mg/kg), However, all values remained below internationally accepted safe limits. The transfer factor values (0.02–0.35) indicated low mobility of metals from the soil to the coffee beans, with Cu being the most mobile of the metals. Metal concentrations in the study areas showed significant differences (p < 0.05) and strong positive correlations between individual metals. The carcinogenic risk (CR) of Cr is 8.7 × 10⁻⁵, which is within the acceptable lifetime risk range as recommended by the USEPA (10^-^⁶ to 10^−4^). The levels of the analyzed heavy metals in green Coffee beans were below the recommended safe levels, which mean that coffee produced in the western Guji zone is safe for human consumption as regards heavy metal contamination. These findings provide valuable input for environmental monitoring, sustainable land management, food safety assessment and quality assurance in specialty coffee from Ethiopia.

## 1 Introduction

Coffee is one of the most important agricultural commodities in the world, supporting the livelihoods of millions of farmers and making a significant contribution to the economies of major producing countries such as Brazil, Vietnam, Colombia and Indonesia [1]. The *Coffea* genus includes over 60 species; however, only coffee *(Coffea arabica* L*)*, *Coffea canephora* (Robusta) and *Coffea* liberica are commercially important, accounting for about 60–70 % of the world coffee production [2]. Ethiopia is widely recognised as a centre of origin and genetic diversity for *C. Arabica*, where a variety of soils are cultivated under favourable agro-ecological conditions, contributing to the production of high quality specialty coffee [3], [4], [5]*.*Coffee has both economic and nutritional value as well as functional value and is a source of antioxidants and other bioactive compounds such as chlorogenic acids, caffeine and phenols, which are associated with a number of health benefits, including antioxidant, inflammatory and anticancer activity [6]. The chemical composition and flavour of coffee beans are highly influenced by genetic factors, climatic conditions, soil properties and cultivation practices [7].Despite its economic and nutritional importance, Coffee production is increasingly threatened by the contamination of agricultural land with heavy metals. Heavy metals are produced by both natural processes such as rock weathering and anthropogenic activities such as mining, industrial emissions, excessive use of fertilisers and pesticides, irrigation effluents and atmospheric deposition [8]. Unlike many organic pollutants, heavy metals are persistent, non-biodegradable and may accumulate in soils and enter the food chain via the uptake of plants. Essential elements such as copper (Cu), zinc (Zn), chromium (Cr), cobalt (Co) and nickel (Ni) are required in trace quantities for normal physiological processes, but excessive concentrations can damage the growth of plants and reduce the quality of crops. Non-essential metals such as cadmium (Cd) and lead (Pb) are toxic even at low concentrations and can accumulate in the tissues of edible plants, including beans [9], [10], [11], [12], [13], [14], [15], [16], [17], [18], [19], [20].Recent studies have reported on the presence of heavy metals in coffee soils and Coffee beans in several coffee producing countries, showing that soil characteristics, environmental conditions and agricultural practices have a strong influence on the bioavailability of metals and on the transfer of metals from soil to plants [21]. Accumulation of toxic metals in coffee beans can change their chemical composition and quality and increase the risk of chronic exposure to humans through regular consumption of coffee. Long-term exposure to high concentrations of toxic metals is associated with adverse health effects, including renal dysfunction, neurological impairment, reproductive toxicity and carcinogenicity.Therefore, the continued monitoring of heavy metals in coffee production systems is essential for ensuring food safety and the respect of international quality standards.Although some studies have reported on the concentration of heavy metals in Ethiopian coffee, limited information is available on the transfer of heavy metals from soil to coffea beans and the potential health risks from *Coffee* consumption in the West Guji *Coffee* Specialized Zone [22]

## 2 Material and methods

### 2.1 Description of study area

The study area was carried out in three locations in the West Guji Zone of Oromia Reginal state, southern Ethiopia. These locations are Hambella Wamena, Bule Hora Wereda, and Kerca Woreda. The study area is approximately 467 km south of Addis Ababa, ranging in latitude from 5°35′ to 5°63′ N and longitude from 38°15′ to 38°25′ E.

### 2.2 Chemicals and reagents

All reagents used in this investigation were analytical grade. Reagents used in the digestion of the samples are: nitric acid (HNO₃, 69–72%), hydrochloric acid (HCl, 37%), and perchloric acid (HClO₄, 70%). The H₂O₂ (30%) solution was used to enhance the oxidative phase during digestion. In all the experiments conducted, deionized water was used for preparing solutions and making their dilutions. Nitrate of metals solutions were used for preparation standard of the following metal, Pb, Cd, Cr, Cu, Mn, Co, and Ni.

### 2.3 Instruments and apparatus

Polyethylene bags were used to dry and store the samples before the analysis. The grinding and pulverization of the samples was done by the use of a ceramic mortar and pestle, and the ashing was done using crucibles (Haldenwanger, Germany). Weighing of the samples was achieved using an analytical balance with an accuracy of 0.0001 g, with the use of a heat shield (Moulinex, France), together with 100 ml digestion tubes fitted with a reflux condenser attached to a heating apparatus (Gallenhamp, England). Volumes of the solutions were measured using borosilicate flasks (25, 50, 100, and 1000 ml), Duran measuring cylinders (Germany), and Pyrex pipettes (USA). The volumetric flasks, measuring cylinders, and digesting tubes were cleaned using detergent and tap water, deionized water, distilled water, nitric acid, then washed again and kept in a clean place.

### 2.4 Experimental methods

#### 2.4.1 Sample collection

The sampling was done during the main season for harvesting *Coffea* beans from November to January 2025. The sample were taken from three important coffee producing districts (Kercha, Bule Hora and Hambela Wamena) of West Guji Zone Southern Ethiopia and have been selected in a rigorous manner due to their importance for coffee production sites. Within these three districts, six (6) representative *Coffea* farms (two per districts) have been selected on the basis of availability, production intensity and willingness of farmers to take part in the project. The farms selected corresponded to the predominant coffee growing conditions in the study area. Each holding comprised between two and four hectares. For Homogeneity of the samples from each site (district), at least five Coffee plants (zigzag sampling pattern) total of ten Coffee plants from one district were selected for sampling of coffee beans. Soil samples were collected near each coffee plant in a distance of 75 cm and depth of 0–20 cm, which represent the root zone where heavy metals can be absorbed which is similar with reported by some modification [23].

#### 2.4.2 Preparation of coffee bean samples

The collected coffee beans underwent cleaning in order to separate them from any dirt, leaves, or twigs and to ensure that only ripe and undamaged beans were included in the batch. After this, the beans were left to dry in the sun for more than four weeks. Once dried, the beans were husked and an amount of 50 g of ground coffee beans was selected for testing purposes.

#### 2.4.3 Preparation of soil samples

The soil samples were treated in the same way as the coffee samples; they were dried in air after removing all plant parts, ground, mixed, and passed through a sieve with a 2 mm mesh size. In every district, more than 1 kg of sieved soil was collected, out of which 50 g was taken for analysis.

#### 2.4.4 Sample digestion of soil

The wet digesting procedure was used to digest the soil sample. A digestion tube was filled with precisely 0.5 g of each of the soil samples that had been air-dried, crushed, and sieved. To adequately mix the sample, 5 ml of aqua regia and 0.5 ml of H_2_O_2_ were measured, put to the digestive tube, and gently stirred. After that, the digestion tubes were put on a digestive furnace (Model: KDN-20C, China) and cooked for three hours at 180°C. After cooling, all of the digests were filtered through Whatman No. 42 filter paper and diluted with double-distilled water to a volume of 50 milliliters. After being digested in five duplicates, each sample was placed in a glass container with a stopper that had been cleaned with acid, labeled, and stored for metal analysis.

#### 2.4.5 Digestion of coffee bean samples

Prior to the analysis of heavy metals, samples of green coffee beans were cleaned, dried in an oven to a constant weight and ground into a fine powder. The wet digestion process has been optimized and used for the preparation of the samples. 1 g of the powdered coffee sample was transferred to the digestion flask and 2 ml of concentrated nitric acid (HNO_3_), 3 ml of per chloric acid (HCl) and 1 ml of hydrogen peroxide (H_2_O_2_) were added to the solution. The mixture was heated on the digestion block at an optimum temperature until clear solutions were obtained. After cooling, the digest was filtered, transferred to a volume flask and diluted with deionized water to the desired volume. The resulting solution was then analyzed for Cu, Zn, Mn, Cr, Ni, Cd, Co and Pb by flame atomic absorption spectroscopy (FAAS).

#### 2.4.6 Preparation of standard concentration solution

The stock standard solutions (1000 mg/kg) of each metal were prepared by dissolving in deionised water sufficient quantities of the relevant nitrate salts. Serial dilutions of these stock solutions were then performed to obtain working calibration standards in the required concentration range for each metal. For the spiking experiments, the mixed standard solutions were prepared by transferring the corresponding volume of the individual stock solution into a volume of 100 ml, in order to reach the following target concentrations (mg/kg): Mn (2.5), Cu (1.5), Cr (1.5), Pb (3.5), Zn (3), Cd (4.5), Cd (4.5), Cq (1.5) and Ni (2.5).

### 2.5 Method validation

Method validation is the procedure of confirming that an analytical method is suitable for its intended use. Parameters used to validate the method were the detection limit, quantification limit, accuracy, and precision.Limit of detection is the lowest concentration of an analyte that can be identified; however it may not be quantifiable with an acceptable degree of uncertainty [19]. To calculate the LOD for each element, the pooled standard deviation (σ) for the blank sample is multiplied by three (3σblank, N = 9) as given in (Eq. 1).

(1) MDL= 3*SD

Where SD, is the Standard deviation of replicate measurements of low level matrices.

The limit of quantification (LOQ) is the minimum concentration of an analyte in a sample that can be accurately measured with acceptable uncertainty [24]. LOQ in this research was obtained directly from triple analyses of each reagent blank sample using Eq. 2.

(2) LOQ= 10*SD

Where SD = Standard deviation of replicate measurements

The accuracy of the analytical approach has been evaluated by means of recovery tests utilizing Laboratory Control Samples (LCS). The known concentrations of target metals were spiked in the pre-digested samples and analysed in the same way as the actual samples. The recovery percent of the samples was calculated using the following equation (Table 1).

(3) $$\text{Recovery }\%\;=\;\frac{\text{concentration Spike– concentration Unspike}}{\mathrm{ammount}\;\mathrm{of}\;\mathrm{added}(\frac{\mathrm{mg}}{L})}\text{x}100\%$$

**Table 1:** Parameters (reagent volume, temperature and time) tested to optimize digestion method employed for coffee bean samples.

Procedure	Mixture of Reagent HNO_3_:HClO_4_:H_2_O_2_ (ml)		Temperature (^°^C)	Time (hrs)		Observation		Remark
Optimization for volume of reagents								
A	4, 5,1		230	3:00		Brown		Prohibited
B	5, 2,1		230	3:00		Brown		Prohibited
C	5,3,1		230	3:00		Yellow		Prohibited
D	3, 4, 1		230	3:00		light-yellow		Prohibited
E	2, 3, 1*		230	3:00		Colorless		Prohibited
Optimization for temperature								
A	2, 3, 1		120	3:00		Brown		Prohibited
B	2, 3, 1		160	3:00		Brown		Prohibited
C	2, 3, 1		180	3:00		Yellow		Rejected
D	2, 3, 1		220	3:00		Light yellow		Prohibited
E	2, 3, 1		230*	3:00		colorless		Selected
Optimization for time								
A	2, 3, 1		230	1:00		Brown		Prohibited
B	2, 3, 1		230	1:30		Brown		Prohibited
C	2, 3, 1		230	2:00		Yellow		Prohibited
D	2, 3, 1		230	2:30		light yellow		Prohibited
E	2, 3, 1		230	3:00*		Colorless		Selected

**Note:** * indicates the optimal volume, temperature and time employed for acid digestion of *Coffee* bean samples

The precision refers to the closeness of agreement between a series of independent measurements obtained under the conditions specified. The precision of an analytical technique can be determined by using the variance, standard deviation, coefficient of variation, and relative standard deviation values of the set of values (Eq. 4) [25].

(4) $$\%\mathrm{RSD}=\;\frac{\mathrm{SD}}{x}x100\%$$

Where: SD = standard deviation of the results X = mean concentration value

The analytical performance of the method developed was assessed with respect to instrumental detection limits (IDL), limit of detection (LOD), limit of quantification (LOQ), accuracy (recovery) and precision (RSD) for heavy metals in Coffee and soil samples. IDL values ranged from 0.005 to 0.050 mg/L, indicating an adequate instrumental sensitivity to detect the metals under investigation at the trace level [26]. LOD values ranged from 0.006 to 0.060 mg/L for Coffee samples, and from 0.001 to 0.007 mg/L for soil samples. Similarly, the LOQ values for the soil samples ranged from 0.020 to 0.200 mg/L of coffee and from 0.010 to 0.030 mg/L of soil. These relatively low detection and quantification limits show that the method is sensitive enough to detect trace metals in both matrices as it was explained in (Table 2), The slightly lower LOD and LOQ values observed in soil samples can be attributed to the difference in matrix composition and the absorption efficiency of the digestion [27].

**Table 2:** Instrumental detection limit, limit of detection and limit of quantification values for the studied heavy metals of Coffee bean and soil sample.

Metal	IDL(mg/L)	MOD(mg/L) for C	MOD(mg/L) for S	MOQ(mg/L) for C	MOQ(for S	%R for C	%R for S	%RSD for C	%RSD for S
Cu	0.02	0.054	0.002	0.180	0.01	115	114	2.64	2.82
Ni	0.04	0.051	0.007	0.170	0.03	87	90	3.11	2.11
Zn	0.005	0.039	0.003	0.130	0.01	98.6	96	3.41	2.83
Co	0.050	0.060	0.003	0.200	0.01	114	110	2.1	1.3
Cd	0.005	0.006	0.001	0.020	0.01	112.8	108	3.65	5.38
Pb	0.010	0.030	0.004	0.100	0.01	94.5	102	1.5	2
Cr	0.050	0.051	0.006	0.17	0.01	91	95	2.32	2.76
Mn	0.010	0.030	0.002	0.095	0.01	92.8	94	1.89	1.1

R = Recovery, C = Coffee, S = soil, RSD = Relative standard deviation,

The method's accuracy, expressed as *a* percentage of recovery (R), ranged from 87% to 115% for samples of Coffee and 90–114% for samples of soil. These recovery values are within the generally acceptable 80–120% range for trace metal analysis [28], which indicates good accuracy of the method and negligible matrix interference. The precision of the method was determined from the reproducibility of triplicate measurements (n = 3) of each metal tested. The %RSD is the measure of precision and its value ranges between 1.1% and 5.38% for all metals that were analyzed in Coffee and soil. All the obtained results are significantly lower than the maximum limit of 15% set as a permissible limit [29].

### 2.6 Quantification of heavy metals

The quantitative analysis of heavy metals has been carried out by Flame Atomic Absorption Spectrophotometry (FAAS). The instrument has been calibrated with a reagent blank and five-point calibration curves based on working standard solutions for each of the analytes. Concentrations of Cu, Pb, Cr, Ni, Cd, Co, Mn and Zn in digested samples were determined using the Buck 210VGP AAS (USA) under optimised instrumentation. Final concentrations of the metals in the soil and Coffee bean samples were estimated using the following formula.

(5) $$c=\frac{B}{W}\mathrm{xV}$$

Where B = mg/kg of metal in digested sample, V = final volume of the digested sample solution (ml) W = weight of digested sample (g). Instrumental conditions for the analysis of the heavy metals using FAAS described in (Supplementary Table 1)

### 2.7 Health risk assessment

The potential health risks associated with consumption of *Coffee* beans containing heavy metals have been assessed using the models of estimated daily intake (EDI), hazard quotient (HQ), hazard index (HI) and carcinogenic risk (CR).

#### 2.7.1 Estimated daily intake (EDI)

Estimated Daily Intake is a measure used in health risk assessment to estimate the amount of a contaminant consumed by a person on a daily basis through food intake in relation to body weight. The standard EDI adult equation is:

(6) $$\mathrm{EDI}=\frac{C\times \mathrm{IR}}{\mathrm{BW}}$$

Where: C = metal concentration (mg/kg, mean), IR = ingestion rate (kg/day), BW = body

Weight (kg),

(7) $$\mathrm{For}\mathrm{child}\;\mathrm{EDI}=\;{\mathrm{EDI}}_{\mathrm{child}}x\frac{70}{15}$$

Where, 70 kg = Adult Body weight, 15 kg = Child body weight

#### 2.7.2 Hazard quotient (HQ)

Hazard Quotient is a risk assessment value used in environmental health and toxicology to estimate the potential for non- carcinogenic adverse health effects of exposure to a chemical, as described in the formula below.

(8) $$\mathrm{QH}=\;\frac{\mathrm{EDI}}{\mathrm{RfD}}$$

Where:

• Estimated Exposure = amount of chemical a person is exposed to
• Reference Dose (RfD) = maximum safe daily exposure level established by regulatory agencies

#### 2.7.3 Hazard index (HI)

Hazard Index is the sum of hazard quotients (HQ) for multiple contaminants or routes of exposure. It is used to assess the overall non- carcinogenic health risk of a combined exposure (Eq. 8).

(8) HI= ∑Hqi

#### 2.7.4 Carcinogenic risk (CR)

Carcinogenic risk is a quantitative estimate of the likelihood that a person will develop cancer over his life as a result of exposure to a carcinogenic agent, as shown in Eq. 9.

(9) CR=EDI x CSF

Where= **EDI** is the estimated daily intake (mg kg⁻¹ day⁻¹) and CSF is the oral cancer slope factor.

### 2.8 Transfer factor (TF)

Transfer factor is the measure of the efficiency of the migration of metals from the soil into the coffee*.* Transfer factor is defined as the ratio of the concentration of the metal in the *Coffee*, expressed as (mg/kg, dry weight), to the concentration of the same metal in the soil, expressed as mg/kg dry weight.

(6) $$\mathrm{TF}=\;\frac{\mathrm{Ccoffee}}{C\;\mathrm{soil}}$$

Where: TF = Transfer Factor

Coffee = Concentration of metal in coffee (mg/kg), dry, weight)

soil= Concentration of metal in soil (mg/kg) dry, weight)

### 2.9 Moisture determination coffee beans

The moisture content of coffee beans was measured using the oven-drying method. A hot air oven set at 105°C was used to dry a sample of ground coffee beans with a known weight until the weight stayed constant. After being dried and allowed to cool in a desiccator, the sample was weighed once again. The difference between the original and final weights was used to calculate the moisture content as a percentage of the initial sample weight. *Coffee* bean quality and storage stability are commonly assessed using this method with some modification [30]

### 2.10 Optimization of digestion procedure of coffee bean

The digestion procedure for coffee bean samples has been optimised to achieve complete decomposition of the organic matrix and effective recovery of target metals. The different acid mixtures (nitric acid, 70%), hydrochloric acid, 70%) and hydrogen peroxide, 30%, digestion temperatures and digestion times have been systematically assessed. The digestion optimized method consisted of 2 ml HNO_3_, 3 ml HClO_4_ and 1 ml H_2_O_2_ at 230 °C for 3 h per 1 g of sample. Optimisation focused on obtaining a clear and colourless solution, minimising the consumption of reagents and ensuring maximum recovery of the analyte.

### 2.11 Optimization of digestion procedure of soil sample

Supplementary table 2 shows the optimized procedure. A 0.5 g sample of dried and ground soil was put in a flask and mixed with aqua regia, which is a mixture of 3 parts HCl (37%) and 1 part HNO_3_ (69–72%), approximately 5 ml in total, and 1.5 ml of H_2_O_2_. The mixture was then heated in sequence on a Kjeldahl apparatus equipped with a condenser, starting at 180 °C for 30 min, then increasing to 240 °C for an additional 30 min, and lastly, 270 °C for 2 h

### 2.12 Instrument calibration and operating conditions

Calibration curves showed excellent linearity and correlation coefficients (R²) ranged from 0.9985 to 0.9989 for all metals examined (Table 3). These values are above the commonly accepted threshold value (R² > 0.995) for analytical methods and confirm that there is a strong linear relationship between the absorbance and the concentration of the analyte [30]. This high level of linearity guarantees a reliable interpolation of the sample concentrations within the calibration limits set. Calibration concentration ranges (mg/L) and linear calibration curve equations (y = mx + b) for the determination of Cu, Mn, Ni, Co, Pb, Cr, Zn, and Cd using FAAS were determined in (Supplementary Fig1).

**Table 3:** Calibration concentration ranges (mg/L), observed correlation coefficients, and calibration curve equation for the metals under study.

Metal	Standard solution concentration (mg/L)	Correlation coefficients	calibration curve equation
Pb	0, 1, 2, 3, 4	r^2^= 0.9989	y = 0.006x - 0.0006
Cu	0, 1, 2, 3, 4	r^2^= 0.9988	y = 0.032x - 0.002
Ni	0, 0.5, 1, 1.5	r^2^= 0.9986	y = 0.003x + 0.0003
Mn	0, 2.5, 4, 5.5	r^2^= 0.9985	y = 0.028x + 0.0015
Zn	0, 2.5, 4, 5.5	r^2^= 0.9988	y = 0.067x + 0.0082
Cd	0, 0.5, 1, 1.5	r^2^= 0.9987	y = 0.028x - 0.001
Cr	0, 2.5, 4, 5.5	r^2^= 0.9985	y = 0.002x - 0.0001
Co	0, 1, 2, 3, 4	r^2^= 0.9986	y = 0.012x + 0.0002

### 2.13 Statistical data analysis

The statistical analysis was carried out using SPSS (version 29.0). One-way ANOVA was used to determine the significant differences in metal concentration between the sampling sites, with significance set at p < 0.05. For the assessment of the strength and direction of the relationship between the metals analysed (Cu, Ni, Zn, Co, Cd, Pb, Mn and Cr), Pearson correlation coefficients were calculated.

## 3 Results and discussion

### 3.1 Moisture determination of coffee samples

In this section, the moisture content of the coffee beans collected from different collection sites, such as Kercha, Bule Hora, and Hambella Wamena, was evaluated. The moisture content values ranged between 8.6% and 9.5%, where the highest value was found in Bule Hora, 9.5%, followed by Hambella Wamena, 8.9%, and then Kercha, 8.6%. The values were found to be well below 11.5%, which is the ideal value for the *c*offee beans to be stored and exported [30]. The differences in the values could be due to the local climatic conditions, drying, and duration of storage. The results showed that the coffee beans are suitable for long-term storage and export (Table 4).

**Table 4:** Percentage (%) of moisture content of coffee bean sample (X ± SD, n = 3).

No.	study area	Moisture (%)
1	Kercha	8.6 ± 0.2
2	Bule hora	9.5 ± 0.4
3	Hambella wamena	8.9 ± 0.3

### 3.2 Concentration levels of selected metals in coffee bean samples

The levels of concentration of multiple metals in coffee bean samples collected from Kercha, Bule Hora, and Hambella Wamena showed considerable differences, suggesting that the composition of the local soils affects the uptake of metals by coffee plants (Table 5, Table 6).

**Table 5:** Mean concentration (X ± SD, n = 3, mg/kg, dry weight) of metals in coffee bean.

Number	Elements	Kercha	Bule hora	Hambella wamena
1	Cu	20.20 ± 1.4	22.44 ± 1.7	20.10 ± 1.3
2	Ni	1.43 ± 0.3	1.34 ± 0.1	1.22 ± 0.2
3	Zn	8.43 ± 1.1	6.74 ± 1.1	6.99 ± 1.2
4	Co	1.47 ± 0.3	1.49 ± 0.2	1.48 ± 0.1
5	Cd	ND	ND	ND
6	Pb	ND	ND	ND
7	Cr	0.92 ± 0.1	0.08 ± 0.01	0.82 ± 0.01
8	Mn	17.14 ± 1.3	14.25 ± 1.2	15.11 ± 1.33

**Table 6:** Mean concentration (X ± SD, n = 3, mg/kg, dry weight) of elements in soil samples.

Number	Elements	kercha	Bule hora	Hambella wamena
1	Cu	59.40 ± 2.3	64.40 ± 2.5	61.54 ± 2.43
2	Ni	7.12 ± 0.8	5.40 ± 0.01	6.77 ± 2.11
3	Zn	40.92 ± 2.0	30.30 ± 2.1	36.21 ± 2.45
4	Co	6.30 ± 0.3	9.33 ± 0.13	7.45 ± 1.1
5	Cd	0.82 ± 0.03	1.01 ± 0.03	0.83 ± 0.02
6	Pb	ND	ND	ND
7	Cr	3.63 ± 0.11	3.55 ± 0.03	3.60 ± 0.01
8	Mn	285.44 ± 6.3	237.11 ± 3.3	250.21 ± 5.1

From the Table 5 showed the metal content in coffee beans from Kercha, Bule Hora, and Hambela Wamena West Guji Zone Oromia regional state, Southern Ethiopia. The concentrations of heavy metals in coffee beans collected from Kercha, Bule Hora, Copper (Cu) was the most common essential trace element, with concentrations ranging from 20.10 ± 1.30–22.44 ± 1.70 mg/kg, followed by manganese (Mn) (14.25 ± 1.20–17.14 ± 1.30 mg/kg) and zinc (Zn) (6.74 ± 1.10–8.43 ± 1.10 mg/kg). While cadmium (Cd) and lead (Pb) were not found in any of the examined *Coffee* bean samples, lower quantities of nickel (Ni) (1.22 ± 0.20–1.43 ± 0.30 mg/kg), cobalt (Co) (1.47 ± 0.30–1.49 ± 0.20 mg/kg), and chromium (Cr) (0.08 ± 0.01–0.10 mg/kg).The variations among the three districts are likely associated with differences in soil geochemistry, parent rock composition, climatic conditions, and agricultural management practices, all of which influence metal uptake by *Coffea* plants [23]. The concentrations of Cu, Zn, Ni, Co, Mn, and Cr were comparable to values reported for coffee beans from other coffee-producing regions and fell within internationally acceptable limits for foodstuffs, suggesting that these vital trace elements are present at normal background levels without excessive accumulation [31] and Furthermore, the absence of detectable Cd and Pb—two of the most toxic and bio accumulative heavy metals—suggests minimal anthropogenic contamination and reflects the relatively unpolluted environmental conditions of the West Guji coffee-growing areas. Significant differences were found for the concentrations of Cu, Ni, Co, Zn, Mn, and Cd, as revealed by the ANOVA results (p < 0.001). From the p-values, it is revealed that these variables are not independent, and there is a significant association between the heavy metal concentrations and the group of coffee beans [24].

### 3.3 Distribution form of metals in soil samples

Table 8 shows the results of the mean concentration (Mean ± SD, n = 3) of certain heavy metals in the soil from Kercha, Bule Hora, and Hambella Wamena. Copper and manganese were the most abundant metals detected in the study area, while lead was not detected. The concentration range of copper varied between 59.40 ± 2.3–64.40 ± 2.5 mg/kg. It can be assumed that this concentration could result from the natural mineralogy of the soil, together with the application of fungicides in agricultural practices. Highest manganese levels were recorded, ranging from 237 ± 3.3–285 ± 6.3 mg/kg. This is characteristic of tropical soils that have a higher manganese content. Zinc levels were recorded at lower levels, ranging from 30 ± 2.1–40 ± 2.0 mg/kg. Nickel levels were recorded at 5 ± 0.01–7 ± 0.8 mg/kg, while cobalt levels were recorded at 6 ± 0.3–9 ± 0.13 mg/kg. Chromium levels were recorded at a stable range of 3.5 ± 0.03–3.6 ± 0.11 mg/kg. Cadmium levels were recorded at 0.82 ± 0.03–1.01 ± 0.03 mg/kg. All the recorded levels were found to be within the safe limits established for the respective metals in the soils of the world. Lead levels were not recorded, while cadmium levels were found to be at safe limits. Therefore, no pollution from human sources is a threat to the environment [32]. Significant variations were recorded in the levels of the metals across the districts under consideration. All the metals showed significant variations at p < 0.05.Although cadmium was found in the soil, it was not found in the coffee beans, and the elements in the coffee beans did not closely relate to those in the soil. This implies that there was little uptake from the soil to the coffee plant. This uptake is affected by various elements other than the total concentration of the metal in the soil, such as solubility, uptake by the plant, and physiological properties of the coffee plant [33]. Soil properties that affect the availability of the metal to the plant include soil pH, texture, cation exchange capacity, organic matter, clay, and redox state [34]. All these factors help to explain the differences in the metal concentration in the soil and Coffee beans, although some of them may appear somewhat ambiguous when combined.

### 3.4 Comparison of the metal contents in coffee bean varieties with other reported values

The metal content in different varieties of coffee beans tends to show similar trends in composition, although variations in content are due to differences in factors such as their origin, soil composition, and processing techniques, as shown in Table 7.

**Table 7:** Comparison of observed metals concentration (mg/kg, dry mass) in green coffee beans with the reported values.

Metal	Present Study	Dubale), [33]	Berego, [35]	Adler, [32]	Omer, [19]	PML	Type of plant	Standard Reference	Status
Cu	20.20–22.44	23.4–28.5	0.14–0.29	14 ± 6.43	NR	30	*in* coffee	[36]	Within safe limit
						40	*in foods*	[37]	
Mn	14.25–17.14	17.3–23.6	0.08–0.11	NR	0.48–28.7	500	*Plants* (Gesho)	[35]	Within safe limit
Ni	1.34–1.43	1.66–2.43	NR	0.42 ± 0.04	0.0–0.25	1.63	EP	[38]	Within Safe limit
						40	*in foods*	[37]	
Pb	ND	NR	NR	0.076 ± 0.096	0.0–23.88	1.00	*in* coffee	[39]	Safe (Not detected)
Zn	6.74–8.43	8.74–12.7	0.054–0.076	3.6 ± 0.6	0.0–4.59	30	*in* coffee	[39]	Within safe limit
						27.4	EP	[40]	
Cd	ND	NR	NR	0.02 ± 0.005	0.00–8.01	1.00	*in* coffee	[36]	Safe (Not detected)
						0.3	MP	[41]	
Cr	0.08–0.09	1.04–1.92	NR	NR	NR	0.10	*in* coffee	[36]	Within safe limit
						2.0	MP	[37]	
Co	1.47–1.49	2.47–2.86	NR	NR	NR	40	*in food*	[42]	Within safe limit
						3	EP	[42]	

MPL = Permissible limit (mg/kg), MP= Medicinal plants, EP = Edible plants, ND = Not detected, NR = Not reported

Table 8 compares the results of this study with internationally recognized safe limits for heavy metals in green coffee beans, which indicates low contamination and good quality. The concentrations of copper (20.20–22.44 mg/kg) and zinc (6.74–8.43 mg/kg) were lower than acceptable limits, but fell in the same range as that reported by Dubale [33], but higher than in Berego [35] due to variation in soil composition, agricultural practices and environmental conditions. The manganese levels (14.25–17.14 mg/kg) were significantly below the maximum authorized limit for plants, so toxicity was not a concern [38]. Nickel concentrations (1.34–1.43 mg/kg) were within the acceptable limits as defined by WHO [42], but close to the reference value and therefore need to be monitored. Most importantly, green *Coffee* beans have shown good safety quality in comparison with Codex and international limits. The results confirm the safety of eating green coffee beans in terms of heavy metal contamination, as all detected metals are within the acceptable limits of FAO and WHO, the Codex Alimentarius Commission and WHO. These differences between studies, such as those of [32], [33] and Omer (2011 [19], can be attributed to geographical origin, analytical methods and post-harvest treatment.

**Table 8:** Pearson correlation coefficients of metals in the green coffee beans.

Metal	Cr	Co	Cu	Cd	Mn	Ni	Zn	Pb
Cr	1.00							
Co	−0.113	1.00						
Cu	−0.287	−0.660	1.00					
Cd	0.532	0.311	0.433	1.00				
Mn	0.513	0.034	0.371	0.247	1.00			
Ni	−0.517	−0.431	0.643	−0.213	−0.210	1.00		
Zn	−0.168	−0.778	0.834	−0.531	0.281	0.714	1.00	
Pb	0.032	0.123	−0.321	0.0124	0.116	0.034	0.015	1.00

### 3.5 Comparisons of metal concentrations to literature values

Soil metal content is significantly influenced by the parent material, mineral composition, and particle size distribution. These parameters combine to produce a wide range of content. In this research, the concentration of Zn (30.30–40.92 mg/kg), Cr (3.55–3.63 mg/kg), and Co (6.30–9.33 mg/kg) in the soils is comparable with the established background levels of Zn (1.80–438 mg/kg), Cr (7–150 mg/kg), and Co (0.1–100 mg/kg. [43]. Manganese levels were found to be within the range of the established background levels for agricultural soils.

The copper (Cu) content varied from 59.40 to 64.40 mg/kg, whereas the cadmium (Cd) content varied from 0.82 to 1.01 mg/kg. The results are in agreement with the copper content ranging from 0.18 to 68.75 mg/kg and cadmium content ranging from 0.21 to 1.02 mg/kg in reference [44]. Lead (Pb), a potentially deleterious metal, was below the method detection limit in all soil samples. This is in agreement with the results of Sultana *et al*. [45].

### 3.6 Pearson correlation of metals in green coffee beans samples

Pearson correlation analysis was conducted to investigate the relationships between the levels of certain metals in green coffee bean samples. Correlation coefficients (r) were derived to measure the relationship between each pair of metals using the method described by [43]. Table 9 below shows the Pearson correlation matrix.

**Table 9:** Correlation matrices for metals in soil sample (n = 3).

**metals**	**Cu**	**Zn**	**Mn**	**Ni**	**Co**	**Cr**	**Cd**
Cu	1						
Zn	0.700	1					
Mn	0.848	0.637	1				
Ni	0.897	0.679	0.867	1			
Co	0.493	0.706	0.377	0.385	1		
Cr	0.717	0.689	0.644	0.787	0.284	1	
Cd	0.810	0.532	0.708	0.805	−0.033	0.783	1

The results obtained from the Pearson correlation analysis for the heavy metals are presented in Table 8. The results show strong positive correlations between Cr and Mn (r = 0.513), Cr and Cd (r = 0.532), Cu and Ni (r = 0.643), Cu and Zn (r = 0.834), and Ni and Zn (r = 0.714). These results indicate that these elements might be originating from a common source or might be behaving similarly. Similarly, moderate positive correlations were found for Co and Cd (r = 0.311), Cu and Mn (r = 0.371), Cd and Mn (r = 0.247), and Zn and Mn (r = 0.281).

However, high negative correlations were found for Cr and Ni (r = −0.517), Co and Cu (r = −0.660), and Co and Zn (r = −0.778). Similarly, moderate negative correlations were found for Cr and Cu (r = −0.287), Co and Ni (r = −0.431), Cu and Pb (r = −0.321), Cd and Ni (r = −0.213), and Mn and Ni (r = −0.210).

These inverse relationships, therefore, suggest that increased levels of one metal could be inhibiting the uptake or translocation of another, most likely due to competitive absorption or antagonistic effects in the plant system. The other metal combinations showed weak positive and negative correlations, implying minimal interaction and possible separate sources and absorption mechanisms. The correlation analysis, therefore, gives an indication of the possible sources, mobility, and interaction mechanisms of the metals in green coffee beans.

### 3.7 Pearson correlation of metals within soil sample

A Pearson correlation analysis was conducted to evaluate the correlation between the concentrations of individual metals present in the soil samples collected from the studied area. Correlation coefficients were calculated to identify the possible sources of the metals, their geochemical associations, and similarities in their retention properties. Table 9 presents the correlation matrix for the three soil samples.

From Table (Table 9) reveals that the correlation between Cu and Mn is significantly high at 0.848, while the correlation between Cu and Ni is 0.897. Similarly, the correlation between Mn and Ni is 0.867, while the correlation between Ni and Cd is 0.805. In addition, Cd is significantly correlated with Cu at 0.810, Mn at 0.708, and Cr at 0.783. All the statistically significant correlations reveal that the metals may have a common lithogenic source or may be affected by anthropogenic activities, which may include agricultural activities, fertilizers, and amendments. In addition, the presence of the metals may be due to similar retention mechanisms. There were moderate positive correlations between Zn and Cu (r = 0.700), Zn and Mn (r = 0.637), Zn and Ni (r = 0.679), Zn and Cr (r = 0.689), Cr and Cu (r = 0.717), and Cr and Mn (r = 0.644). In contrast, the element Co showed relatively low correlations with the majority of the metals. It showed moderately positive correlations with Cu (r = 0.493) and Zn (r = 0.706), while it showed low correlations with Mn (r = 0.377), Ni (r = 0.385), and Cr (r = 0.284). There was a slight negative correlation between Co and Cd at a correlation coefficient of −0.033. Thus, the presence of positive correlations among the majority of the metals suggests that the distribution of the metals is significantly influenced by the physicochemical properties of the soil. These interactions between the soil and the metals may be reflected in the bioavailability and uptake of the metals into the coffee plants growing in the area [46].

### 3.8 Soil–coffee transfer

The transfer factors show that there is limited absorption of essential elements and restricted mobility of hazardous metals. The positive correlations between the concentration of metals in coffee and soil indicate that the composition of the soil plays a major role in the accumulation of metals. When the transfer factor (TF) value is greater than 1, it shows that there is a high accumulation and transfer of metals from the soil to the coffee beans. When the transfer factor (TF) value is less than 1, it shows that there is restricted uptake and mobility of metals from the soil to the coffe*e* beans. To evaluate the accumulation of metals in crops and human exposure to metal uptake through dietary intake, the transfer factor (TF) is usually use.The soil-Coffee transfer from each sites was tabulated in (Table 10).

**Table 10:** Soil-to-Coffee transfer factor (TF) of selected heavy metals.

No.	Element	Kercha TF	Bule Hora TF	Hambella Wamena TF	Mean TF
1	Cu	0.34	0.35	0.33	0.34
2	Ni	0.20	0.25	0.18	0.21
3	Zn	0.21	0.22	0.19	0.21
4	Co	0.23	0.16	0.20	0.20
5	Cd	ND	ND	ND	ND
6	Pb	ND	ND	ND	ND
7	Cr	0.25	0.02	0.23	0.17
8	Mn	0.06	0.06	0.06	0.06

TF = Transfer Factor (dimensionless); ND = Not detected in coffee beans.

The heavy metals' transfer factors (TF) from the soil to the coffee beans in the West Guji Zone were generally below unity. Copper showed a moderate transfer factor (mean TF = 0.34), and it could be concluded that copper is a nutrient element readily absorbed by the *Coffee* plants and well within the physiological safety limits. Zinc, nickel, and cobalt showed low to moderate transfer factors (0.16–0.25), well within the regulated bioaccumulation process, as influenced by the properties of the soil and the metabolic processes of the plants. Manganese, despite its high concentration in the soil, showed the lowest value of the transfer factors (0.06), indicating its strong retention properties in the soil. Chromium, as a result of its low bioavailability in the soil, caused by adsorption and complexation, showed a low value of the transfer factor (mean TF = 0.17) which is similar with [25].The absence of cadmium (Cd) and lead (Pb) in coffee beans, as shown by the results where TF = ND, indicates that coffee have limited uptake of these two elements and effective exclusion mechanisms. The values of the transfer factor (TF) for the various heavy metals, ranging from 0.02 to 0.35, indicate a low probability of the accumulation of heavy metals by coffee grown in the region of the study as it was observed in Table10. The uptake of the heavy metals by the *Coffe*a plants, arranged in order of uptake, is as follows: Copper > Zinc ≈ Nickel ≈ Cobalt > Chromium > Manganese. Since the values of the transfer factors are less than unity, it indicates that there is a limited uptake of the heavy metals by the coffee plants in the West Guji Zone. This implies a reduced risk of the heavy metals entering the coffee product, a desirable outcome for the environmental safety of the coffee production system.

### 3.9 Health risk assessment for heavy metals through Coffee consumption

In particular, toxic metals of public health interest like Cd and Pb were found to be below the detection limits for all the samples. This minimizes any potential exposure concerns. The results of the health risk assessment revealed that the exposure to heavy metals associated with the consumption of green coffee beans harvested from Kercha, Bule Hora, and Hambella Wamena regions would be minimal. This is because the concentrations of the metals measured, i.e., Cu, Mn, Zn, Ni, Co, and Cr, was below the internationally accepted maximum allowable levels of these metals in the food products. Therefore, there is no significant risk to human health. However, the results showed that the toxic metals of public health concern, i.e., Cd and Pb, were below the detection limits for the samples. This implies that the exposure to these metals would be minimal, and there would be no significant impact on human health. Therefore, the results of the study showed that the green coffee beans harvested from the regions of interest would be safe for human consumption [30]

Cn= concentration, a= adult, c= child, b= both adult and child, NE= Not established, ND = Not Detected, NA = Not Applicable, CSFs= oral cancer slope factors, CR= Carcinogenic risk, RFD = Reference Dose, HQ = Hazard Quotient *Pb has no safe RFD; value shown is a screening reference level (USEPA). From Table 11, shows estimated daily intakes (EDI), hazard quotient (HQ), hazard index (HI) and carcinogenic risk (CR) values for adults and children. EDI values for all metals tested were below their respective oral reference doses (RFDs), suggesting relatively low dietary exposure from Coffee consumption. Among the analyzed metals, copper exhibited the highest EDI for both adults (5.97 ×10⁻³ mg kg⁻¹ day⁻¹) and children (2.79 ×10⁻² mg kg⁻¹ day⁻¹), followed by zinc, cobalt, nickel, and chromium. Cadmium and lead were not detected in the coffee bean samples; therefore, their estimated daily intake was considered negligible. The hazard quotient (HQ) for each individual metal was below one in adults, ranging from 0.01 for Zn to 0.15 for Cu, suggesting that consumption of Coffee beans is unlikely to result in any adverse non- carcinogenic effects on health in adults. The cumulative hazard index (HI) for adults was 0.26, all related data’s Adopted Oral Reference Dose (RfD) Values and Derivation Parameters for Heavy Metals Used in Health Risk Assessment were explained (Supplementary Table 3 and 4) well below the safety threshold of 1, further demonstrating that there are no significant non-cancerary health risks associated with long-term coffee use as it was reported [47]

**Table 11:** *Coffee* bean–based health risk assessment for heavy metals in West Guji Zone.

Metal	Mean Cn (mg/kg)	EDI^a^ (mg/kg·day)	EDI^c^	RfD^b^ (mg/kg·day)	HQ^a^	HQ^c^	Oral CSF^b^ (mgL⁻¹day⁻¹)	CR^a^	CR^c^
Cu	20.91	5.97 × 10⁻³	2.79 × 10⁻²	0.04	0.15	0.70	NE	NA	NA
Ni	1.33	3.80 × 10⁻⁴	1.77 × 10⁻³	0.02	0.02	0.09	NE	NA	NA
Zn	7.39	2.11 × 10⁻³	9.85 × 10⁻³	0.30	0.01	0.03	NE	NA	NA
Co	1.48	4.23 × 10⁻⁴	1.97 × 10⁻³	0.02	0.02	0.10	NE	NA	NA
Cr	0.61	1.74 × 10⁻⁴	8.12 × 10⁻⁴	0.003	0.06	0.27	0.5	8.7 × 10⁻⁵	4.06 × 10⁻⁴
Cd	ND	0	0	0.001	0	0	NE	NA	NA
Pb	ND	0	0	0.0035*	0	0	6.1	NA	NA
(HI)	—	—		—	0.26	1.19	NE	—	—

In children, the individual metal HQ values were also below one, with copper (HQ = 0.70) and chromium (HQ = 0.27) accounting for the highest proportion of the total non- carcinogenic risk. However, the cumulative hazard index (HI) was 1.19, which is slightly higher than the recommended safety threshold of 1. This result suggests that although no single metal individually presents a significant non-carcinogenic risk, combined exposure to several metals may pose a potential health problem in children due to their lower body weight and higher exposure per kg body weight. Consequently, children are an especially vulnerable group and continuous monitoring of coffee heavy metal concentrations is recommended. Carcinogenic risk assessment was performed only for chromium because an oral cancer slope factor is available for this element. The carcinogenic risk (CR) for adults was 8.70 × 10⁻⁵, which falls within the acceptable lifetime cancer risk range of 10⁻⁶ to 10⁻⁴, indicating an acceptable carcinogenic risk associated with coffee consumption. In contrast, the estimated carcinogenic risk for children was 4.06 × 10⁻⁴, exceeding the generally accepted upper limit of 10⁻⁶ to 10⁻⁴. This finding suggests that prolonged exposure to chromium through coffee consumption could pose a potential carcinogenic concern for children under the assumed exposure scenario, emphasizing the importance of minimizing heavy metal contamination in coffee-producing areas and implementing routine monitoring programs to ensure consumer safetyas it was tabulated in (Table 11).A carcinogenic risk assessment has been carried out only for chromium, as an oral carcinogenicity factor is available for this element. The carcinogenic risk (CR) in adults was 8.70 × 10⁻⁵, which is within the acceptable lifetime cancer risk range of 10⁻⁶ to 10⁻⁴, indicating that coffee consumption is associated with an acceptable carcinogenic risk. On the other hand, the estimated carcinogenic risk in children was 4.06 × 10⁻⁴, which is higher than the generally accepted upper limit of 10⁻⁶ to 10⁻⁴. This finding indicates that long-term exposure to chromium via coffee consumption could be a potential carcinogenic concern for children in the exposure scenario assumed and underlines the importance of minimizing heavy metal contamination in coffee production areas and of carrying out routine monitoring programmes to ensure consumer safety.

## 4 Conclusions

The study assessed the concentration, distribution and transfer of selected heavy metals (Cu, Mn, Zn, Co, Cr, Cd and Pb) in coffee beans and soils, from the districts of Kercha, Bule Hora and Hambale Wamena in the West Guji Zone of Ethiopia. The results showed that the predominant metals in both the soil and coffee samples were basic metals, in particular copper and manganese, while toxic metals such as cadmium and lead were not detected in the *coffee* beans. While the cumulative hazard index (HI = 1.19) for children marginally exceeded the safe limit, suggesting a possible non-carcinogenic health risk from long-term exposure, the estimated daily intake (EDI) and hazard quotient (HQ) values for all detected metals were below the acceptable safety threshold (HQ < 1) for adults. Continuous monitoring of heavy metal contamination is advised to reduce potential health hazards, especially for children, even though the carcinogenic risk (CR) for chromium remained below the allowed range (10⁻¹–10⁻¹) for adults and was at the highest tolerable limit for children. All the detected concentrations of metals in coffee beans were within internationally accepted acceptable limits, which confirmed the safety and quality of the *Coffee* produced in the area of study. Based on the findings of this study, Regular assessment of the mechanisms for transferring soil and plants, together with regular assessment of health risks, is essential to protect consumers, in particular vulnerable groups such as children. it is strongly recommended that monitoring of the concentration of heavy metals in coffee-growing soils and Coffee beans be continued in order to ensure environmental sustainability, food safety and compliance with international quality standards. Farmers should be encouraged to adopt sustainable farming practices, including the prudent use of fertilizers, pesticides and soil-altering measures to avoid toxic metal accumulation in the future. Soil management strategies should be encouraged to maintain optimal pH, organic matter content and nutrient balance, thereby reducing the bioavailability of metals and the uptake of metals by plants. In addition, future studies should examine seasonal variations, metal speciation and bioavailability, and the effects of post-harvest processing on metal concentrations.

## Author contributions

• **Yakob Geda Debeli** conceived and designed the experiments, performed the experiments, analyzed the data, prepared figures and/or tables, authored or reviewed drafts of the article, and approved the final draft.

• **Gemechu Duguma Argessa** conceived and designed the experiments, analyzed the data, authored or reviewed drafts of the article, and approved the final draft.

• **Dr. Tola Jabessa Masho** conceived and designed the experiments, analyzed the data, authored or reviewed drafts of the article, and approved the final draft.

• **Mizbawu Kenea Gurmu** performed the experiments, authored or reviewed drafts of the article, and approved the final draft

• **Derejje Abdisa Saketa** authored or reviewed drafts of the article, and approved the final draft

• **Kenenisa Abdisa Kuse** performed the experiments, authored or reviewed drafts of the article.

## CRediT authorship contribution statement

**Gemechu Duguma Argessa:** Investigation, Formal analysis, Data curation. **Tola Jabessa Masho:** Writing – review & editing, Validation, Formal analysis. **Yakob Geda Debeli:** Writing – review & editing, Writing – original draft, Validation, Supervision, Software, Methodology, Investigation, Formal analysis, Data curation, Conceptualization. **Saketa Derejje Abdisa:** Formal analysis, Data curation, Conceptualization. **Gurmu Mizbawu Kanea:** Supervision, Investigation, Conceptualization. **Kenenisa Abdisa Kuse:** Writing – review & editing, Formal analysis, Data curation.

## Funding

This research did not get any specific grant from funding agencies in the public, commercial, or not-for-profit sectors.

This research was financially supported by an internal research grant from the Research Directorate, Bule Hora University, Ethiopia

## Declaration of Competing Interest

The authors declare the following financial interests/personal relationships which may be considered as potential competing interests. Yakob Debeli reports administrative support was provided by Bule Hora University, Ethiopia. Reports a relationship with that includes: Has patent pending to. If there are other authors, they declare that they have no known competing financial interests or personal relationships that could have appeared to influence the work reported in this paper.

## Data Availability

Data will be made available on request.
