# Supplementary material for: Determination of selected heavy metals in soil and their impact on coffee bean composition, in case of West Guji Zone, Ethiopia
Source: Toxicol Rep. 2026 Aug 8;17:102323. doi: 10.1016/j.toxrep.2026.102323 (PMC13520102; doi:10.1016/j.toxrep.2026.102323)
Supplement: Supplementary file 1 [file mmc1.docx]

**Supplementary Table1. Instrumental conditions (wave length, silt width, lamp and lamp current) for the analysis of the heavy metals using FAAS.**

| Metals | **Cu** | **Ni** | **Zn** | **Co** | **Cd** | **Pb** | **Mn** | **Cr** |
| --- | --- | --- | --- | --- | --- | --- | --- | --- |
| Wave length (nm) | 324.7 | 232 | 213.9 | 253.65 | 228.9 | 217.0 | 279.48 | 359.35 |
| Slit width (nm) | 0.7 | 0.7 | 0.7 | 0.7 | 0.7 | 0.7 | 0.7 | 0.7 |
| Lamp | HCL | HCL | HCL | HCL | HCL | HCL | HCL | HCL |
| Lamp current (mA) | 1.5 | 2 | 2 | 2 | 2 | 3 | 2 | 2 |

**Supplementary Table2. Different conditions tested for optimization of digestion procedure for 0.5 g soil samples.**

|  | Reagent(s) used | Reagent volume (mL) | Temp.  (^0^C) | Digestion time (hr) | Observation | Remark | |
| --- | --- | --- | --- | --- | --- | --- | --- |
| **Optimization f or reagent volume** | | | |  |  |  | |
| 1 | Aqua-regia:H_2_O_2_ | 4.5:1 | 270 | 3:00 | Deep yellow | Rejected |  |
| 2 | Aqua-regia:H_2_O_2_ | **4:1.5*** | 270 | 3:00 | Light yellow | Selected |  |
| 3 | Aqua-regia:H_2_O_2_ | 4.5:2 | 270 | 3:00 | Light yellow | Rejected |  |
| **Optimization f or temperature** | | | |  |  |  | |
| 1 | Aqua-regia:H_2_O_2_ | 4:1.5 | 240 | 3:00 | Deep yellow | Rejected | |
| 2 | Aqua-regia:H_2_O_2_ | 4:1.5 | **270*** | 3:00 | Light yellow | Selected | |
| 3 | Aqua-regia:H_2_O_2_ | 4:1.5 | 300 | 3:00 | Light yellow | Rejected | |
| **Optimization for digestion time** | | | |  |  |  | |
| 1 | Aqua-regia:H_2_O_2_ | 4:1.5 | 270 | 2:00 | Deep Yellow | Rejected | |
| 2 | Aqua-regia:H_2_O_2_ | 4:1.5 | 270 | 2:30 | Light yellow | Rejected | |
| 3 | Aqua-regia:H_2_O_2_ | 4:1.5 | 270 | **3:00*** | Light yellow | Selected | |

* Shows the optimal condition for the given parameter, Temp = temperature, hr = hour

## Supplementary Table3. Assumptions used in the health risk assessment

| Parameter | Value | Source Convention |
| --- | --- | --- |
| Daily coffee consumption (IR) | 0.02 kg day⁻¹ (≈20 g coffee beans/day) | Commonly used for coffee risk studies |
| Body weight (BW) | 70 kg (adult) | USEPA default |
| Exposure frequency (EF) | 365 days year⁻¹ | Continuous exposure |
| Exposure duration (ED) | 30 years | Adult exposure |
| Averaging time (non-cancer) | ED × 365 = 10,950 days | USEPA |
| Averaging time (cancer) | 70 × 365 = 25,550 days | Lifetime |

$$EDI=\frac{C\times IR}{BW}$$

**Where:**
C = metal concentration (mg kg⁻¹, mean)

IR = ingestion rate (kg day⁻¹)

BW = body weight (kg)

**Estimated Daily Intake (EDI) For copper metal (Cu)**

= $\frac{\mathbf{20.91 x 0.02kgday}\mathbf{-1}}{\mathbf{70 kg (adult)}}$

= 0.00597 or 5.97x10^-3^

Then use the same procedure for all metal: Ni, Pb, Co, Cd, Mn, Zn, and Cr

Ingestion Rate (IR): is the average mass of coffee beans consumed per person per day (kg day⁻¹).

Define coffee consumption pattern

A realistic and commonly accepted assumption in exposure studies is:

- Coffee powder used per cup: 10 g
- Average consumption: 2 cups person⁻¹ day⁻¹

Step 1: Daily coffee intake=10g/cup×2cups/day=20g/day

Step 2: Convert grams to kilograms

20 g/day=0.02 kg/day, Final IR value used: IR=0.02 kg day^−1^

**Reference Dose (RfD): definition and derivation**

General equation used by regulatory agencies

$$RFD= \frac{NOAEL}{UF x MF}$$

**Important:** RfD is **not calculated from data**; it is a **toxicological constant** derived from NOAEL/LOAEL studies and adopted from USEPA/WHO

Where:

- **NOAEL** = No Observed Adverse Effect Level (mg kg⁻¹ day⁻¹)
- **UF** = Uncertainty factor (typically 10–1000)
- **MF** = Modifying factor (usually 1)

RfD represents the **maximum daily oral exposure unlikely to cause adverse health effects over a lifetime**.

**Supplementary Table 4. Adopted Oral Reference Dose (RfD) Values and Derivation Parameters for Heavy Metals Used in Health Risk Assessment**

| Metal | NOAEL (mg kg⁻¹ day⁻¹) | UF | Calculated RfD (mg kg⁻¹ day⁻¹) | Regulatory basis |
| --- | --- | --- | --- | --- |
| Cu | 4.0 | 100 | 0.04 | USEPA |
| Ni | 2.0 | 100 | 0.02 | USEPA |
| Zn | 30.0 | 100 | 0.30 | USEPA |
| Co | 0.03 | 100 | 0.0003 | USEPA |
| Cr (VI) | 0.3 | 100 | 0.003 | USEPA |
| Mn | 14.0 | 100 | 0.14 | USEPA |
| Cd | — | — | 0.001* | USEPA |
| Pb | — | — | No safe RfD* | USEPA |

**NOAEL** = No Observed Adverse Effect Level (mg kg⁻¹ day⁻¹), UF = Uncertainty Factors, RfD = Oral Reference Dose

**Example RfD calculation For Copper (Cu):**

RfD _Cu =_ $\frac{NOAEL}{UF}=\frac{4}{100}=$	0.04mg^-1^Kgday^-1^

**Hazard Quotient (HQ)** as:

QH= $\frac{EDI}{RfD}$

QH for Cu $\frac{0.00597 mg kg⁻¹day⁻¹}{0.04mg kg⁻¹day⁻¹}$^=^ **0.15**

- HQ < 1 → no non-carcinogenic risk
- HQ ≥ 1 → potential health concern

**Hazard Index (HI) calculation**

The Hazard Index (HI) represents the combined non-carcinogenic risk from exposure to multiple metals through coffee consumption.

HI=∑Hqi

**HI calculation**

HI=HQ_Cu_+HQ_Ni_+HQ_Zn_+HQ_Co_+HQ_Cr_+HQ_Mn_=0.15+0.02+0.007+0.02+0.06+0.03 = **0.26**

**HI=0.26<1**

This indicates a **moderate cumulative non-carcinogenic risk**, all metals pose negligible risk (HQ <1).

**Carcinogenic risk (CR) calculation**

As regards carcinogenic risk (CR), it should be noted that not all metals, except chromium, have been shown to have carcinogenic cancer susceptibility factors (CCSFs). Based on the USEPA risk assessment methodology, the carcinogenic risk can only be calculated for metals for which oral CSF is available. The lifetime carcinogenic risk shall be calculated by applying: CR=EDI x CSF

where **EDI** is the estimated daily intake (mg kg⁻¹ day⁻¹) and **CSF** is the oral cancer slope factor.

For chromium (Cr):

- EDI = 1.74×10^−4^ mg kg⁻¹ day⁻¹
- Oral CSF (Cr) = 0.5 (mg kg⁻¹ day⁻¹)
- CR=(1.74×10^−4^) x (0.5=8.7×10^−5^)

**CR _Chromium_ = 8.7×10^−5^**

Pb

| (a) concentration (mg/L) | Absorbance |
| --- | --- |
| 0 | 0 |
| 1 | 0.012 |
| 2 | 0.024 |
| 4 | 0.049 |
| 6 | 0.073 |
| 7 | 0.083 |

Cu

| (b) concentration (mg/L) | Absorbance |
| --- | --- |
| 0 | 0 |
| 0.5 | 0.015 |
| 1 | 0.031 |
| 2 | 0.057 |
| 4 | 0.124 |
| 6 | 0.192 |

Ni

| (c) concentration (mg/L) | Absorbancee |
| --- | --- |
| 0 | 0 |
| 0.4 | 0.002 |
| 2 | 0.007 |
| 3 | 0.01 |
| 4 | 0.014 |
| 7 | 0.024 |
| 8 | 0.027 |

Co

| (d) concentration (mg/L) | Absorbance |
| --- | --- |
| 0 | 0 |
| 1 | 0.012 |
| 2 | 0.024 |
| 4 | 0.049 |
| 6 | 0.073 |
| 7 | 0.083 |

Zi

| (f) concentration(mg/L) | Absorbance |
| --- | --- |
| 0 | 0 |
| 0.5 | 0.046 |
| 2 | 0.147 |
| 4 | 0.278 |
| 5 | 0.334 |
| 6 | 0.408 |

Mn

| (d) concentration(mg/L) | Absorbance |
| --- | --- |
| 0 | 0 |
| 0.5 | 0.013 |
| 2 | 0.052 |
| 4 | 0.105 |
| 6 | 0.166 |

Cd

| (e) concentration(mg/L) | Absorbance |
| --- | --- |
| 0 | 0 |
| 0.4 | 0.008 |
| 0.6 | 0.017 |
| 2 | 0.056 |
| 4 | 0.112 |

Cr

| ( f) concentration.mg/L | Absorbance |
| --- | --- |
| 0 | 0 |
| 2 | 0.0037 |
| 4 | 0.008 |
| 6 | 0.012 |

**Supplementary Fig 1.** Calibration concentration ranges (mg/L), and calibration curve equation for the metals (Cu, Mn, Ni, Co, Pb, Cr, Zn, and Cd) under study
